# Supplementary material for: A Meiotic B-type Cyclin Selectively Drives Mitotic Progression and Fidelity in Cancer Cells
Source: bioRxiv. 2026 Sep 28:2026.09.28.754947. Preprint. [Version 1] doi: 10.64898/2026.09.28.754947 (PMC13648654; doi:10.64898/2026.09.28.754947)
Supplement: Supplementary file 1 [file NIHPP2026.09.28.754947v1-supplement-1.pdf]

bioRxiv preprint doi: https://doi.org/10.64898/2026.09.28.754947; this version posted September 28, 2026. The copyright holder for this preprint (which was not certified by peer review) is the author/funder, who has granted bioRxiv a license to display the preprint in perpetuity. It is made available under a CC-BY 4.0 International license.

Kizhedathu et al, Figure S1

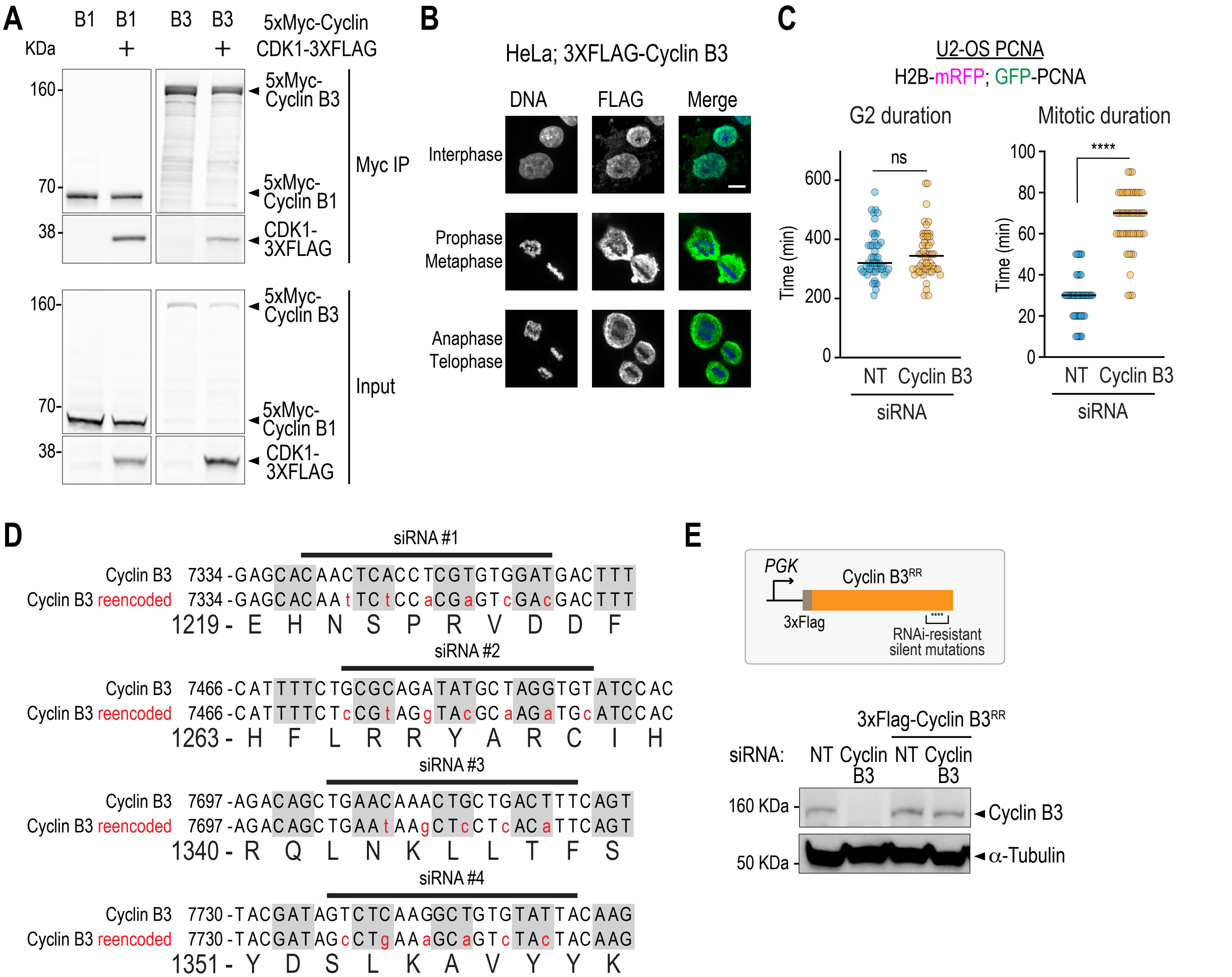

bioRxiv preprint doi: https://doi.org/10.64898/2026.09.28.754947; this version posted September 28, 2026. The copyright holder for this preprint (which was not certified by peer review) is the author/funder, who has granted bioRxiv a license to display the preprint in perpetuity. It is made available under a CC-BY 4.0 International license.

SUPPLEMENTARY FIGURE LEGENDS

Figure S1. Characterization of cyclin B3 interaction with CDK1 and cell cycle phenotypes. ***Related to Fig. 1 and Fig. 2.***

(A) Plasmids encoding for CDK1-3XFLAG were co-transfected into HEK293T cells, along with either 5xMyc-cyclin B1 or 5xMyc-cyclin B3. Cells were subsequently collected and lysed and complexes were pulled down using anti-Myc beads. Note that, under these conditions, 5xMyc-cyclin B1 was more efficient at co-immunoprecipitating CDK1-3XFLAG compared with 5xMyc-cyclin B3, which suggests that cyclin B3 is weaker at interacting with CDK1 compared to cyclin B1. **(B)** Immunofluorescence images of HeLa cells expressing 3xFlag-cyclin B3 and stained with DAPI (blue), α-FLAG (Green) and illustrating examples of cyclin B3 localization in interphase (top), prometaphase and metaphase (middle) and anaphase and telophase (bottom). The images are maximum intensity projections of 4 confocal slices. Note that cyclin B3 localizes to the nucleus in interphase and is largely cytosolic during mitosis. Scale bar, 10 μm. **(C)** Quantification of the duration of G2 *(left)* and mitosis *(right)* phases of the cell cycle from movies of U2-OS cells expressing GFP-PCNA and H2B-mRFP, under the specified conditions. **(D)** Schematics illustrating the binding sites for the cyclin B3 siRNA pool used in this study, as well as the silent mutations engineered in our cyclin B3 reencoded construct (red residues). **(E)** *(top)* Schematic illustrating the construct used for the expression of 3xFlag-tagged, RNAi-resistant (RR) cyclin B3 transgenes. *(bottom)* Immunoblot showing cyclin B3 expression under the indicated conditions. α-Tubulin serves as a loading control. **** represents $P < 0.0001$ from Mann-Whitney tests; non-significant (n.s.) is $P > 0.05$.

bioRxiv preprint doi: https://doi.org/10.64898/2026.09.28.754947; this version posted September 28, 2026. The copyright holder for this preprint (which was not certified by peer review) is the author/funder, who has granted bioRxiv a license to display the preprint in perpetuity. It is made available under a CC-BY 4.0 International license.

Kizhedathu et al, Figure S2

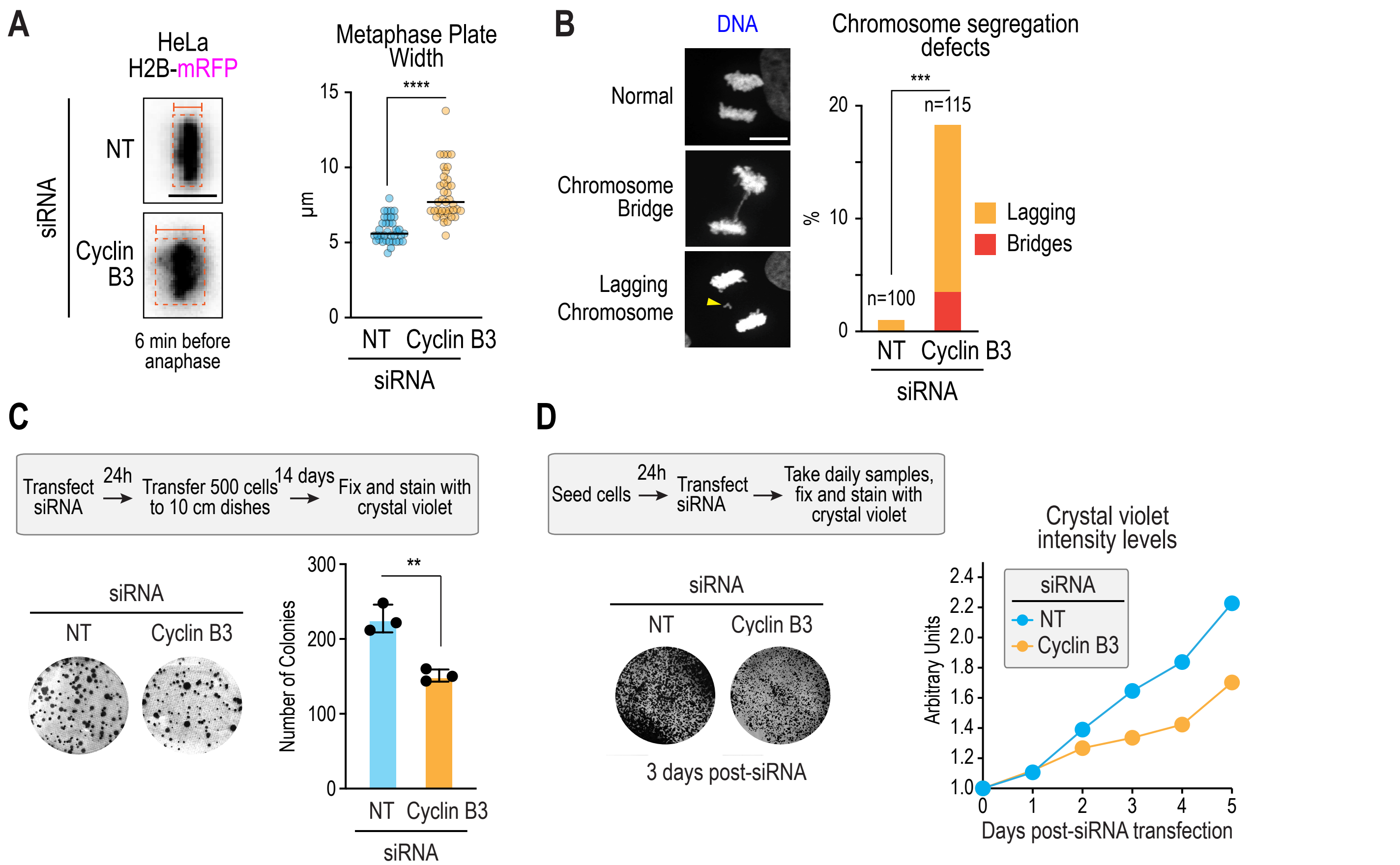

bioRxiv preprint doi: https://doi.org/10.64898/2026.09.28.754947; this version posted September 28, 2026. The copyright holder for this preprint (which was not certified by peer review) is the author/funder, who has granted bioRxiv a license to display the preprint in perpetuity. It is made available under a CC-BY 4.0 International license.

Figure S2. Characterization of cyclin B3 depletion phenotypes. ***Related to Fig. 2.***

(A) *(left)* Representative time-lapse sequences of HeLa cells expressing mRFP-tagged histone H2B and captured 6 minutes before anaphase onset. The red box shows a minimum bounding box encompassing all chromosomes. *(right)* Quantification of metaphase plate width by measuring the width of the minimal bounding box for the specified conditions. Scale bar, 10 μm. **(B)** *(left)* Representative images of anaphase cells stained with DAPI (grey) and showing either chromosome bridges or lagging chromosomes (arrowhead). *(right)* Quantification of chromosome segregation defects for the indicated conditions. Scale bar, 10 μm. **(C)** *(top)* Description of the experimental conditions for the scoring of colony formation. *(bottom left)* Example images of colonies formed by HeLa cells treated with either non-targeting siRNA (NT) or cyclin B3 siRNA. *(bottom right)* quantification of colony numbers under the specified conditions. Error bars are standard deviation. **(D)** *(top left)* Description of the experimental conditions used for the scoring of cell proliferation. *(bottom left)* Example images of cells treated for 3 days with either NT siRNA or cyclin B3 siRNA and stained with crystal violet. *(right)* Growth curves of cells under the specified conditions. For these assays, the crystal violet intensities were scored for each day post-transfection and normalized against values at t=0 days. Each data point represents the average of 6 replicates. Graphs from panels *A* and *C* were analyzed using Mann-Whitney tests; panels *B* was analyzed using Chi square analysis. **** represents $P < 0.0001$; *** represents $P < 0.001$; ** represents $P < 0.01$; non-significant (n.s.) is $P > 0.05$.

bioRxiv preprint doi: https://doi.org/10.64898/2026.09.28.754947; this version posted September 28, 2026. The copyright holder for this preprint (which was not certified by peer review) is the author/funder, who has granted bioRxiv a license to display the preprint in perpetuity. It is made available under a CC-BY 4.0 International license.

Kizhedathu et al, Figure S3

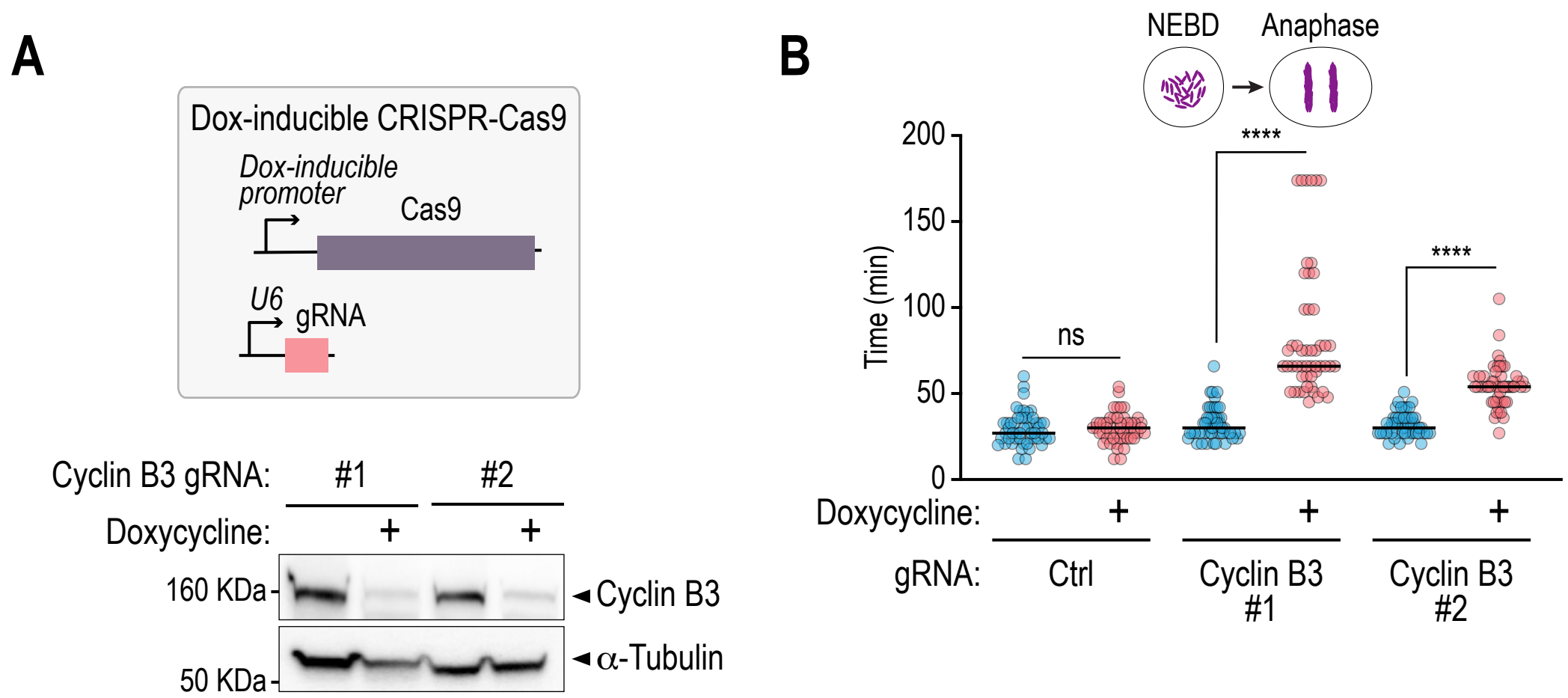

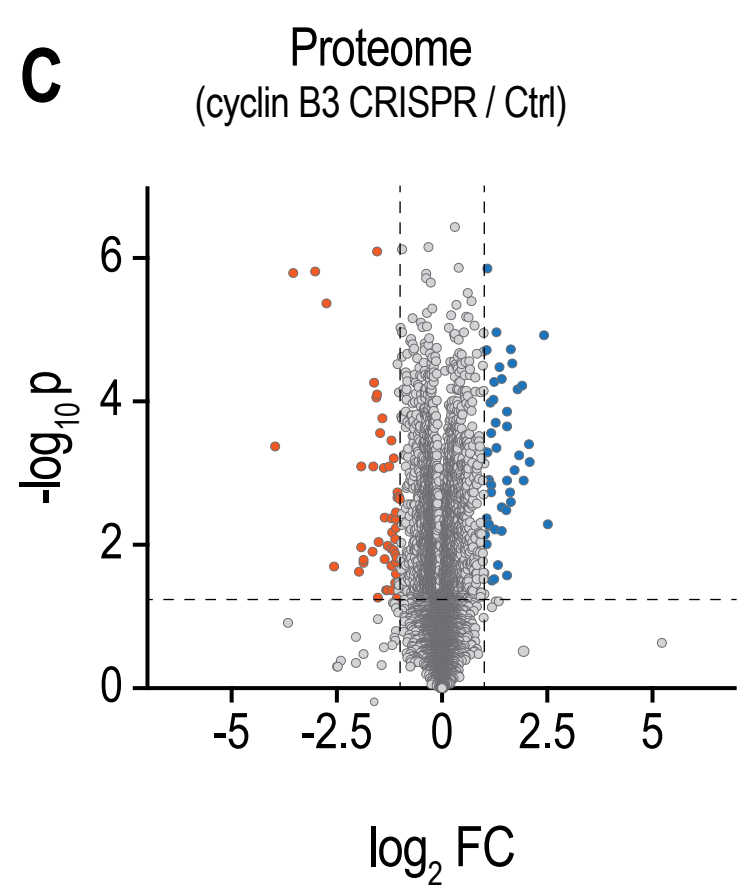

D

<u>Upregulated:</u>
- 220 increased in cyclin B3 CRISPR ($p < 0.05$)
- 920 detected in cyclin B3 CRISPR and either undetected or detected in only one Ctrl replicate

Total: 1,140

<u>Downregulated:</u>
- 341 reduced in cyclin B3 CRISPR ($p < 0.05$)
- 1,187 detected in controls and either undetected or detected in only one cyclin B3 CRISPR replicate

Total: 1,528

bioRxiv preprint doi: https://doi.org/10.64898/2026.09.28.754947; this version posted September 28, 2026. The copyright holder for this preprint (which was not certified by peer review) is the author/funder, who has granted bioRxiv a license to display the preprint in perpetuity. It is made available under a CC-BY 4.0 International license.

Figure S3. Generation and validation of doxycycline-induced CRISPR-Cas9 cell lines for mass spectrometry analyses. *Related to Fig. 3.*

(A) *(top)* Schematic illustrating the constructs used for the generation of doxycycline-induced CRISPR-Cas9-mediated editing of cyclin B3. *(bottom)* Immunoblot showing cyclin B3 depletion upon induction of cyclin B3 guide RNAs (gRNA). α-Tubulin serves as a loading control. **(B)** Quantification of the timing from NEBD to Anaphase onset for the specified conditions. **** represents $P < 0.0001$ from Mann-Whitney tests; non-significant (n.s.) is $P > 0.05$. **(C)** Volcano plot showing whole proteome changes upon cyclin B3 depletion through CRISPR-Cas9 (gRNA #1). Note that only a small number of proteins either increase or decrease upon cyclin B3 depletion. **(D)** Summary of differentially enriched phosphorylated peptides upon cyclin B3 depletion through CRISPR-Cas9. **** represents $P < 0.0001$ from Mann-Whitney tests; non-significant (n.s.) is $P > 0.05$.

bioRxiv preprint doi: https://doi.org/10.64898/2026.09.28.754947; this version posted September 28, 2026. The copyright holder for this preprint (which was not certified by peer review) is the author/funder, who has granted bioRxiv a license to display the preprint in perpetuity. It is made available under a CC-BY 4.0 International license.

Kizhedathu et al, Figure S4

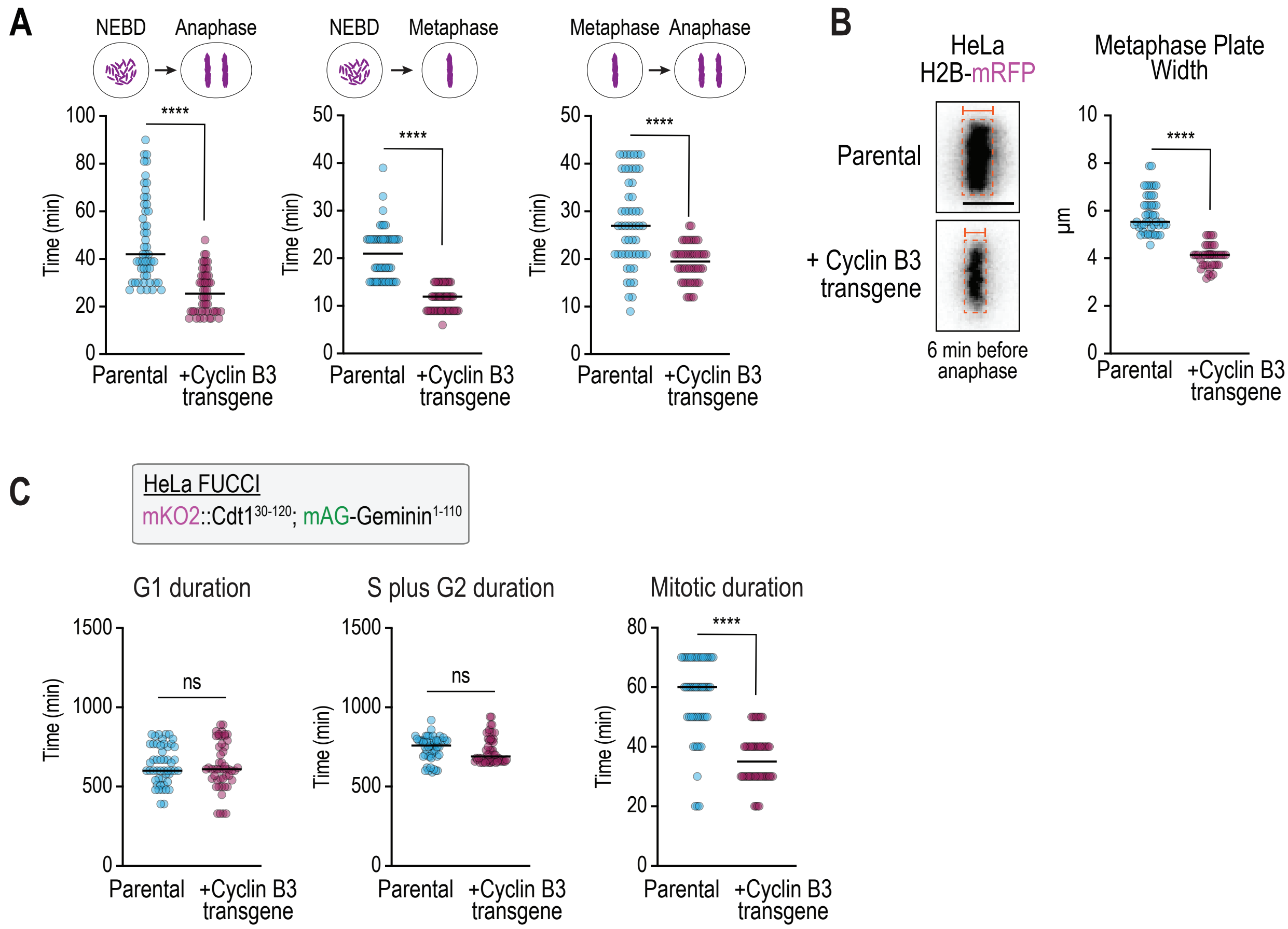

bioRxiv preprint doi: https://doi.org/10.64898/2026.09.28.754947; this version posted September 28, 2026. The copyright holder for this preprint (which was not certified by peer review) is the author/funder, who has granted bioRxiv a license to display the preprint in perpetuity. It is made available under a CC-BY 4.0 International license.

Figure S4. Characterization of the cyclin B3 overexpression phenotype. ***Related to Fig. 4.***

(A) Quantification of the interval from NEBD to Anaphase *(left)*, NEBD to Metaphase *(middle)* and Metaphase to Anaphase *(right)* for the indicated conditions. **(B)** *(left)* Representative time-lapse sequences of HeLa cells expressing mRFP-tagged histone H2B and captured 6 minutes before anaphase onset. The red box shows a minimum bounding box encompassing all chromosomes. Scale bar, 10 µm. *(right)* Quantification of metaphase plate width by measuring the width of the minimal bounding box for the specified conditions. **(C)** Quantification of the duration of G1, S/G2 and mitosis in HeLa cells expressing the FUCCI marker. **** represents $P < 0.0001$ from Mann-Whitney tests;; non-significant (n.s.) is $P > 0.05$.

bioRxiv preprint doi: https://doi.org/10.64898/2026.09.28.754947; this version posted September 28, 2026. The copyright holder for this preprint (which was not certified by peer review) is the author/funder, who has granted bioRxiv a license to display the preprint in perpetuity. It is made available under a CC-BY 4.0 International license.

Kizhedathu et al, Figure S5

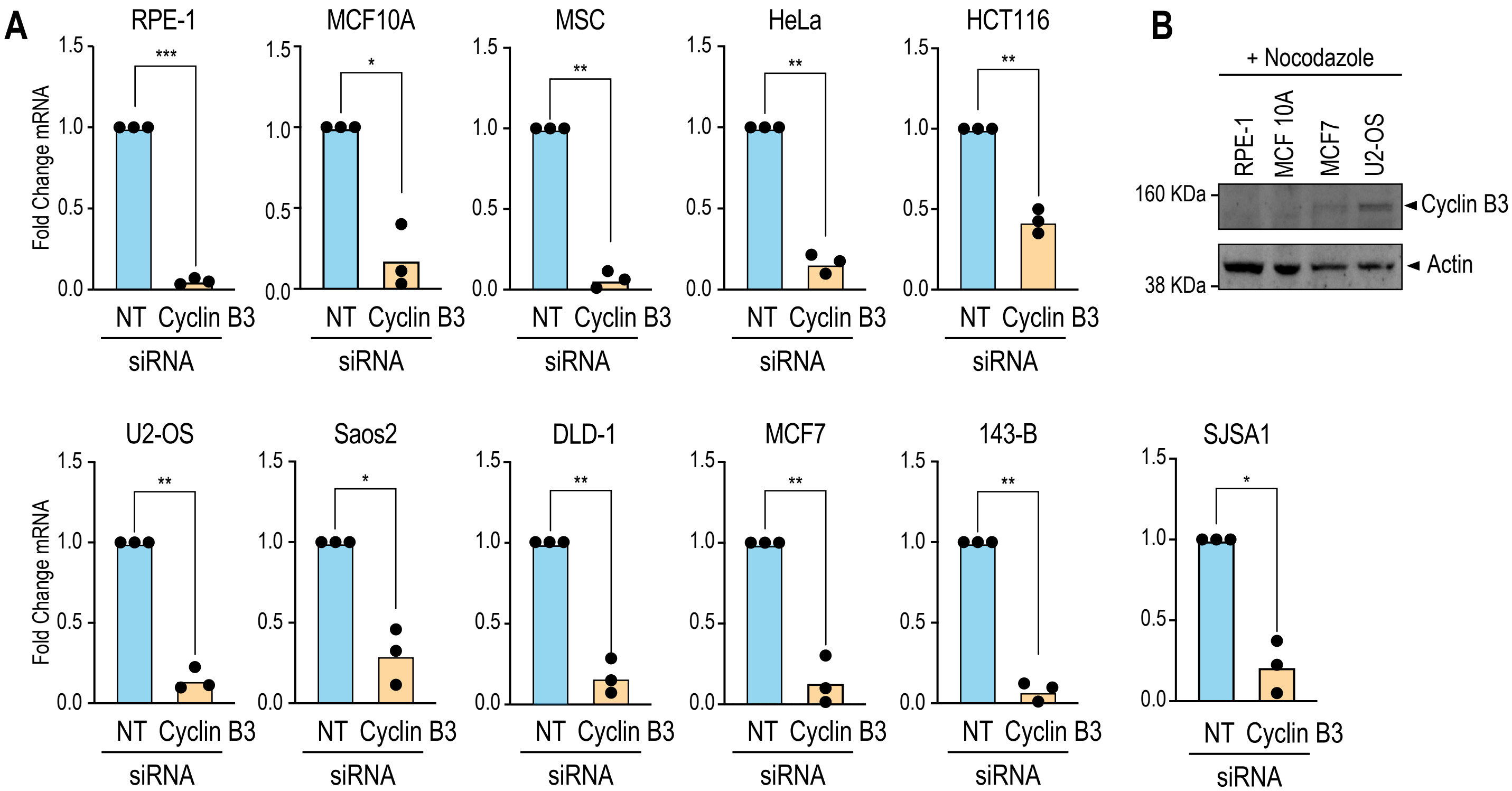

bioRxiv preprint doi: https://doi.org/10.64898/2026.09.28.754947; this version posted September 28, 2026. The copyright holder for this preprint (which was not certified by peer review) is the author/funder, who has granted bioRxiv a license to display the preprint in perpetuity. It is made available under a CC-BY 4.0 International license.

Figure S5. Expression of cyclin B3 in cancer cell lines and validation of cyclin B3 depletion through quantitative PCR (qPCR). ***Related to Fig. 4.***

(A) Quantification of cyclin B3 mRNA levels in cells treated with either NT siRNA or cyclin B3 siRNA. Note that all tested cell lines showed efficient cyclin B3 depletion at the mRNA level. **(B)** Immunoblot showing cyclin B3 expression in mitotically arrested RPE-1, MCF 10A, MCF7 and U2-OS cells. Actin serves as a loading control. **** represents $P < 0.0001$ from Mann-Whitney tests; *** represents P < 0.001; ** represents $P < 0.01$; * represents $P < 0.05$; non-significant (n.s.) is $P > 0.05$.
